# Active Dissolution and Localized Corrosion Behavior of AISI 316L Stainless Steel in Concentrated Hydrochloric Acid

**DOI:** 10.3390/ma19163386

**Published:** 2026-08-09

**Authors:** Citlalli Gaona-Tiburcio, Erick Maldonado-Bandala, Jesús Manuel Jáquez-Muñoz, Demetrio Nieves-Mendoza, Ce Tochtli Méndez-Ramírez, Jose Cabral-Miramontes, Laura Landa-Ruiz, Miguel Ángel Baltazar-Zamora, Luis Daimir Lopez-Leon, Javier Olguin-Coca, Facundo Almeraya-Calderón

**Affiliations:** 1 Universidad Autónoma de Nuevo León, FIME Centro de Investigación e Innovación en Ingeniería Aeronáutica (CIIIA), San Nicolás de los Garza 66455, Mexico; citlalli.gaonatbr@uanl.edu.mx (C.G.-T.); jose.cabralmr@uanl.edu.mx (J.C.-M.); facundo.almerayacld@uanl.edu.mx (F.A.-C.); 2 Facultad de Ingeniería Civil, Universidad Veracruzana, Xalapa 91000, Mexico; erimaldonado@uv.mx (E.M.-B.); dnieves@uv.mx (D.N.-M.); lalanda@uv.mx (L.L.-R.); mbaltazar@uv.mx (M.Á.B.-Z.); 3 Instituto Tecnológico de Ciudad Juárez, Metal-Mechanics Department, Av. Tecnológico 1340, Ciudad Juárez 32500, Mexico; 4 Área Académica de Ingeniería y Arquitectura, Universidad Autónoma del Estado de Hidalgo, Carretera Pachuca-Tulancingo Km. 4.5, Hidalgo 42082, Mexico; luis_lopez@uaeh.edu.mx (L.D.L.-L.); olguinc@uaeh.edu.mx (J.O.-C.)

**Keywords:** localized corrosion, cyclic potentiodynamic polarization, pitting, austenitic stainless steels

## Abstract

AISI 316L austenitic stainless steel is extensively used in petrochemical storage and processing equipment because of its excellent corrosion resistance. However, exposure to concentrated hydrochloric acid severely destabilizes its passive film, promoting active dissolution and localized corrosion. This work investigates the corrosion behavior of AISI 316L stainless steel in hydrochloric acid solutions of 7.2, 9.6, and 12 M at room temperature. Cyclic potentiodynamic polarization (CPP) tests were performed according to ASTM G61, and the corrosion morphology was characterized using optical microscopy (OM), scanning electron microscopy (SEM), energy-dispersive spectroscopy (EDS), and metallographic cross-sections. The electrochemical results revealed an active dissolution regime characterized by the absence of a stable passive region and positive hysteresis loops in all HCl solutions, indicating irreversible surface damage and poor repassivation. The corrosion current density increased from the order of 10^−1^ mA cm^−2^ in 7.2 and 9.6 M HCl to the order of 10^1^ mA cm^−2^ in 12 M HCl, demonstrating a significant acceleration of the corrosion kinetics. SEM and cross-sectional analyses confirmed the development of localized pitting corrosion, with pit depths reaching approximately 0.87 mm. The results demonstrate that concentrated hydrochloric acid promotes the coexistence of generalized active dissolution and localized pitting corrosion, while increasing HCl concentration modifies the morphology and propagation mode of the pits.

## 1. Introduction

The application of stainless steel materials in the manufacture of equipment for the petrochemical industry is in high demand, given the much more demanding application environment in this sector, and the current requirements for these types of alloys are greater in terms of corrosion resistance [1,2,3,4,5].

Stainless steels (SSs) are iron-chromium [Fe-Cr] alloys (minimum chromium content of 11%); the addition of chemical elements to the composition of stainless steels plays an important role in providing various characteristics, and each type is different. Nickel (Ni) provides ductility, but the main objective is to induce an austenitic microstructure, and the presence of molybdenum (Mo) increases hardness to help improve resistance to localized corrosion [6,7]. These steels have diverse applications. Stainless steels are classified according to their microstructure and precipitation into ferritic, martensitic, austenitic, duplex, and precipitation-hardenable (PH) [8,9]. Austenitic stainless steels have a wide range of industrial applications and are often exposed to various corrosive environments, such as industrial, urban, marine, and mixed atmospheres [10,11].

The formation of an oxide layer on the surface of steel depends on the amount of chromium, making it crucial. This layer, rich in chromium oxide (Cr_2_O_3_), is thin (nanometers thick) and is commonly known as a passive film in stainless steels. It is responsible for providing exceptional corrosion resistance [12,13,14,15,16]. When the passive layer is damaged, chemical passivation treatment is used to reform it rapidly, making it adherent, continuous, and compact. Exposure to various corrosive atmospheres can degrade mechanical properties such as fatigue strength and load-bearing capacity [17,18,19,20], thus requiring control and protection methods such as passivation.

It is well-known that aggressive ions, specifically chloride ions (Cl^−^), affect the protective passive film in stainless steels, causing its breakdown. This produces localized attack, such as pitting corrosion [21,22]. The literature review revealed that studies on the corrosion behavior of stainless steel have focused on the corrosion kinetics of passive and transpassive regions, localized corrosion, pitting nucleation, and corrosion mechanisms. DC and AC electrochemical techniques have been the most widely used, including potentiodynamic polarization (PP), cyclic potentiodynamic polarization (CPP), and electrochemical impedance spectroscopy (EIS), respectively, as well as electrochemical noise (EN) in recent decades. Obtaining important electrochemical parameters, such as the passivation range, pitting potentials, corrosion rates, and transpassive regions, is studied using potentiodynamic polarization (PPC) curves.

Several investigations by Krawczyk et al., Mohammed and Ogheneblorhie, He et al., and Wang et al. specifically examined the corrosion behavior of AISI 316L stainless steel in hydrochloric acid solutions because of its widespread use in the chemical and petrochemical industries [23,24]. These studies demonstrated that increasing HCl concentration progressively destabilizes the chromium-rich passive film, promoting active dissolution, localized corrosion, and significant increases in corrosion current density [25]. Previous investigations employed electrochemical techniques such as potentiodynamic polarization, cyclic potentiodynamic polarization, electrochemical impedance spectroscopy, gravimetric measurements, and surface characterization by SEM to evaluate the corrosion kinetics, passive film degradation, pit initiation, and effectiveness of corrosion inhibitors [26]. Collectively, these studies have significantly improved the understanding of corrosion mechanisms in hydrochloric acid media and established the relationship between electrolyte aggressiveness and passive film breakdown [27].

In addition to studies specifically devoted to AISI 316L stainless steel, numerous investigations have examined corrosion mechanisms in other stainless steels exposed to chloride-containing environments using complementary electrochemical techniques.

In addition to the studies specifically conducted on AISI 316L stainless steel in hydrochloric acid solutions, complementary investigations on other stainless steels have contributed to understanding passive film degradation, localized corrosion mechanisms, pit nucleation, and electrochemical behavior in chloride-containing environments. Suresh and Mudali [28] used electrochemical noise to determine the corrosion behavior of AISI 304 austenitic stainless steel immersed in ferric chloride (FeCl_3_). The results indicated that to understand the pitting corrosion mechanism, it would be necessary to relate time-domain data (statistical analysis, noise resistance (Rn), and localization index (LI)) to frequency-domain data through power spectral densities (PSDs). In 2018, Lara et al. [29] studied corrosion behavior using electrochemical noise and potentiodynamic polarization in precipitation-hardened (PH) steels passivated in citric acid as an environmental alternative; the results indicated that the passivation films formed differed with the various passivation acids used. Other investigations [30,31,32,33] used electrochemical techniques such as potentiodynamic polarization and electrochemical impedance spectroscopy to characterize the oxide film through charge transfer processes in passivated 304 SS; the electrochemical parameters of different stainless steels have also been studied by varying pH concentrations in aerated solutions, resulting in oxidizing solutions that promote the formation of protective films, which improve corrosion resistance. Torres et al. [34] determined the influence of tungsten on crevice corrosion in superduplex stainless steels. Potentiodynamic polarization scan (PD), galvanostatic polarization (GS), and reverse potentiodynamic polarization techniques were used to estimate the critical crevice repassivation temperature through tests at different temperatures. The results showed that tungsten improved crevice corrosion resistance at higher initiation and repassivation temperatures, ranging from 7.5 to 15 °C. In 2026, Macedo et al. [35] analyzed the corrosion resistance of UNS S32760 superduplex stainless steel coupons in the presence of hydrochloric acid, with and without an inhibitor (an organic thiourea-based inhibitor), aiming to simulate the conditions of the oil and natural gas industry. Gravimetric tests demonstrated that the addition of the inhibitor reduced the mass loss of the samples when the inhibitor with the highest thiourea concentration was used. Furthermore, electrochemical potentiodynamic polarization tests confirmed the results obtained by gravimetry.

Corrosion is a major problem affecting most industries worldwide, causing significant economic losses, particularly in oil and natural gas processing. The corrosion process not only affects the mechanical and chemical properties of metal alloys, but also alters their physical properties [36,37]. In 2013, the global cost of corrosion was estimated at US$2.5 trillion, representing between 3% and 4% of global GDP [33]. The United States has the world’s largest refining capacity, producing more than 18 million barrels of refined petroleum products per day [38]. According to the National Association of Corrosion Engineers (NACE), the annual cost of corrosion in U.S. refineries exceeds US$3.7 billion [39].

In the petrochemical industry, corrosion occurs in ISO tanks used for transporting and storing chemicals, hydrocarbons, acids, solvents, and process water under potentially highly corrosive conditions. ISO tanks are commonly made of stainless steel and tend to develop localized pitting corrosion due to the presence of hydrochloric acid [40].

Although previous investigations have considerably improved the understanding of stainless steel corrosion in hydrochloric acid environments, most studies have focused on corrosion rates, inhibitor performance, passive film degradation, or electrochemical behavior under specific experimental conditions. Comparatively fewer studies have systematically investigated the active dissolution behavior of AISI 316L stainless steel under highly concentrated hydrochloric acid conditions while correlating cyclic potentiodynamic polarization with detailed pit morphology obtained from SEM and metallographic cross-sectional analysis. Furthermore, limited information is available regarding the influence of increasing HCl concentration on the transition from passive film breakdown to active dissolution under severe acidic environments. The present work addresses this knowledge gap by providing an integrated electrochemical and microstructural evaluation of AISI 316L stainless steel exposed to concentrated hydrochloric acid solutions.

The aim of this work was to investigate the active dissolution and localized corrosion mechanisms of AISI 316L austenitic stainless steel exposed to concentrated hydrochloric acid solutions (7.2, 9.6, and 12 M) at room temperature. Cyclic potentiodynamic polarization (CPP) tests, performed according to ASTM G61, were combined with optical microscopy (OM), scanning electron microscopy (SEM), energy-dispersive spectroscopy (EDS), and metallographic cross-sectional analysis to correlate the electrochemical response with pit morphology and propagation. The study addresses corrosion problems encountered in industrial equipment operating in highly acidic chloride-containing environments and provides insight into the corrosion mechanisms governing AISI 316L stainless steel under extreme acidic conditions.

## 2. Materials and Methods

### 2.1. Materials

The commercial stainless steel employed was an austenitic SS type AISI 316L obtained from a sheet with dimensions 12″ × 12″ and a thickness of 1/8″. The AISI 316L stainless steel, with its ultra-low carbon content, is a variant of 316 SS. Its excellent corrosion resistance makes it an exceptional choice for containers and piping in the petrochemical industry. It is an austenitic steel with excellent corrosion resistance, becomes slightly magnetic after significant cold working, is highly ductile and formable, and exhibits high mechanical strength and hardness at cryogenic temperatures. The addition of molybdenum is responsible for its superior corrosion resistance. One disadvantage of AISI 316L SS is that it cannot be hardened by heat treatment [40]. Chemical composition was determined by the X-ray fluorescence technique (Olympus Scientific Solutions Americas, Waltham, MA, USA), see Table 1.

The samples for the corrosion testing of AISI 316L stainless steel were prepared using metallographic techniques based on ASTM E3. These were ground with silicon carbide (SiC) abrasives with particle sizes ranging from 400 to 800. Subsequently, they were cleaned for 10 min with ultrasound (ethanol) and deionized water [41].

### 2.2. Electrolytes

Hydrochloric acid solutions of 7.2, 9.6, and 12 M were prepared using analytical-grade HCl and distilled water. Municipal tap water supplied by the public drinking water distribution system of Monterrey, Nuevo León, Mexico, was used as the reference electrolyte. According to the official annual water quality report issued by Servicios de Agua y Drenaje de Monterrey (July 2023–June 2024), the water exhibited an average pH of 7.8, turbidity of 1.8 NTU, total dissolved solids of 346 mg/L, total hardness of 292.43 mg/L as CaCO_3_, and sulfate concentration of 162.38 mg/L. These physicochemical characteristics are reported to improve the reproducibility of the experimental conditions [42].

### 2.3. Microstructural Characterization

The microstructure of AISI 316L stainless steel was obtained (prepared by metallography) by scanning electron microscopy (SEM, JSM-5610LV, JEOL Ltd., Akishima, Tokyo, Japan). At different magnifications, backscattered and secondary electron detectors were used to investigate the surface morphology of AISI 316L stainless steel samples subjected to corrosion testing. Elemental analysis was performed using energy-dispersive X-ray spectroscopy (EDS). The dimensions of the pitting on AISI 316L stainless steel after corrosion testing were examined in cross-section using optical microscopy (OM).

### 2.4. Electrochemical Technique

Corrosion tests were performed on AISI 316L stainless steel to evaluate its electrochemical behavior under active dissolution conditions using cyclic potentiodynamic polarization (CPP). Specimens with an exposed working area of 1.0 cm^2^, defined by the corrosion cell, were immersed in tap water and hydrochloric acid solutions at three different concentrations: 12 M (37 wt.% HCl, concentrated hydrochloric acid commonly used in laboratory, chemical, and petrochemical applications), 9.6 M (30 wt.% HCl), and 7.2 M (24 wt.% HCl) at room temperature (25 ± 2 °C).

**Figure 1:**
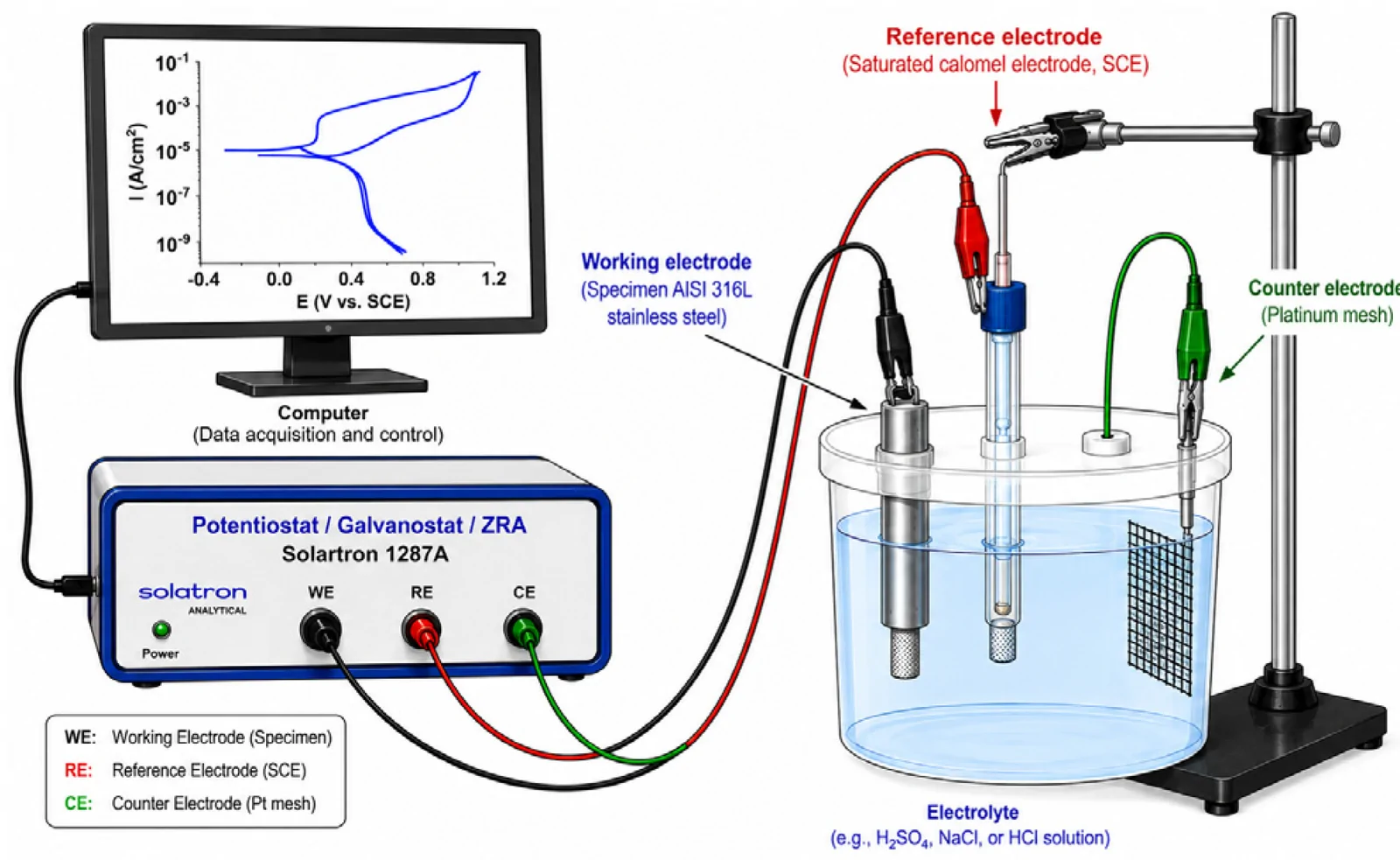
Three-electrode cell for the corrosion measurements.

A conventional three-electrode electrochemical cell was employed using AISI 316L stainless steel as the working electrode (WE), a saturated calomel electrode (SCE) as the reference electrode (RE), and a platinum mesh as the counter electrode (CE) (Figure 1). Electrochemical measurements were performed using a potentiostat/galvanostat/ZRA (Model 1287A, Solartron Analytical, Bognor Regis, West Sussex, UK).

Electrochemical testing was carried out in accordance with ASTM G5-13 and ASTM G61-86 [43,44,45]. Prior to polarization, the open-circuit potential (OCP) was monitored for 50 min until a stable potential was achieved (see Section 3.3.1). Subsequently, cyclic potentiodynamic polarization tests were performed using a potential scan range of ±1000 mV vs. SCE for tap water and ±600 mV vs. SCE for the HCl solutions, at a scan rate of 60 mV min^−1^. All electrochemical tests were conducted in duplicate.

## 3. Results and Discussion

### 3.1. Microstructure by SEM

**Figure 2:**
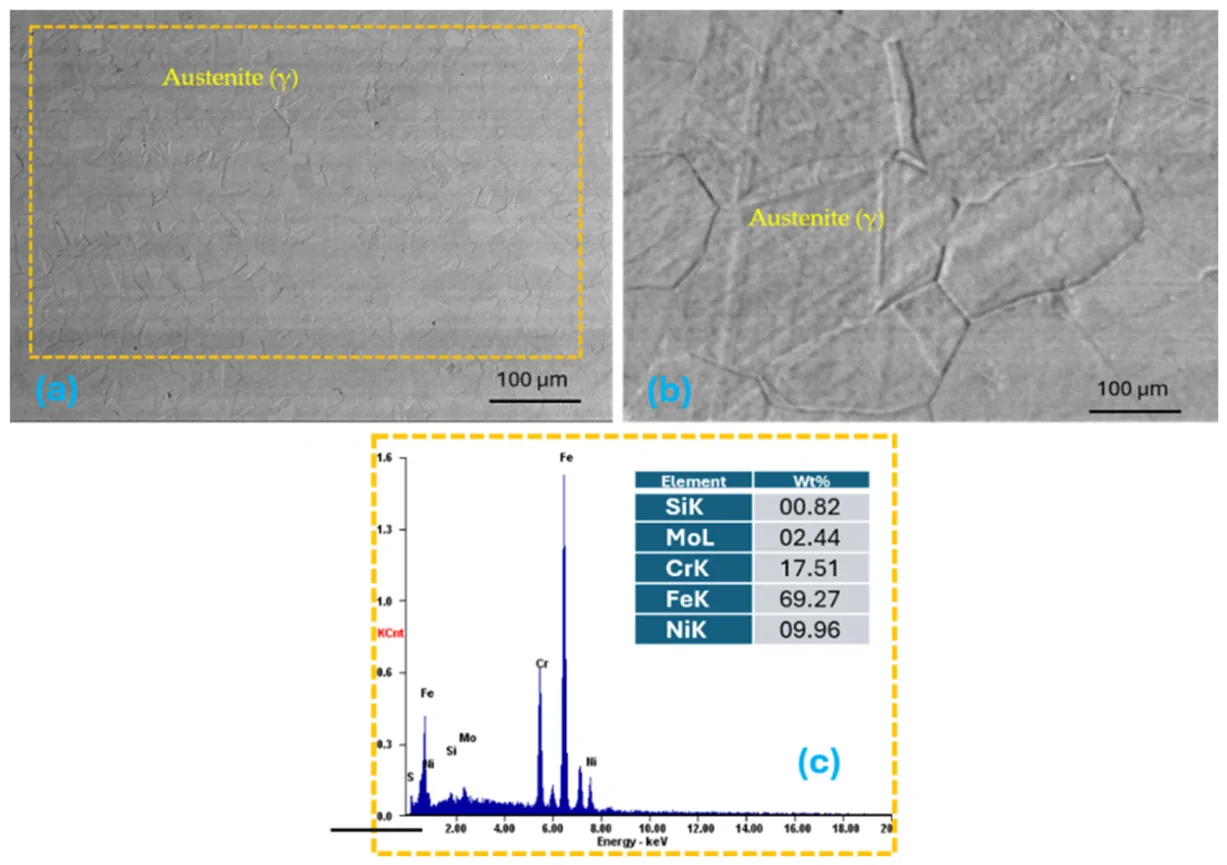
SEM-BES microstructure of AISI 316L SS. (**a**) 200 x, (**b**) 1500 x, and (**c**) EDS spectrum.

The microstructures of AISI 316L SS were analyzed by SEM and consisted primarily of austenite (γ) as the predominant phase (see Figure 2a,b), with equiaxed austenite grains, annealing twins, and well-defined grain boundaries. This microstructure contributed to the austenitic phase exhibiting a face-centered cubic (FCC) crystal structure, high ductility, high toughness, and excellent weldability. Figure 2c shows the energy-dispersive X-ray spectrum, where the elemental composition was obtained, identifying the characteristic elements of AISI 316L SS as iron, chromium, nickel, molybdenum, and silicon, respectively.

### 3.2. Pitting Resistance Equivalent Number (PREN)

In stainless steels, pitting corrosion resistance can be determined using the pitting corrosion equivalent number (PREN). This is an empirical parameter that depends on the steel’s chemical composition. The pitting corrosion resistance of different grades of stainless steel can be determined [46,47,48]. In the literature, the PREN for AISI 316L SS is between 24 and 27. Higher PREN values indicate greater corrosion resistance. It is important to note that essential alloying elements, such as chromium, nitrogen, and molybdenum, determine the PREN (Equation (1)). The PREN result for AISI 316L SS was 24.5 (see Table 2), indicating better pitting corrosion resistance than other steels such as 304 stainless steel (whose theoretical values are between 18 and 20).

PREN = %Cr + 3.3 (%Mo) + 16%N(1)

### 3.3. Electrochemical Measurements

#### 3.3.1. Open Circuit Potential (OCP)

**Figure 3:**
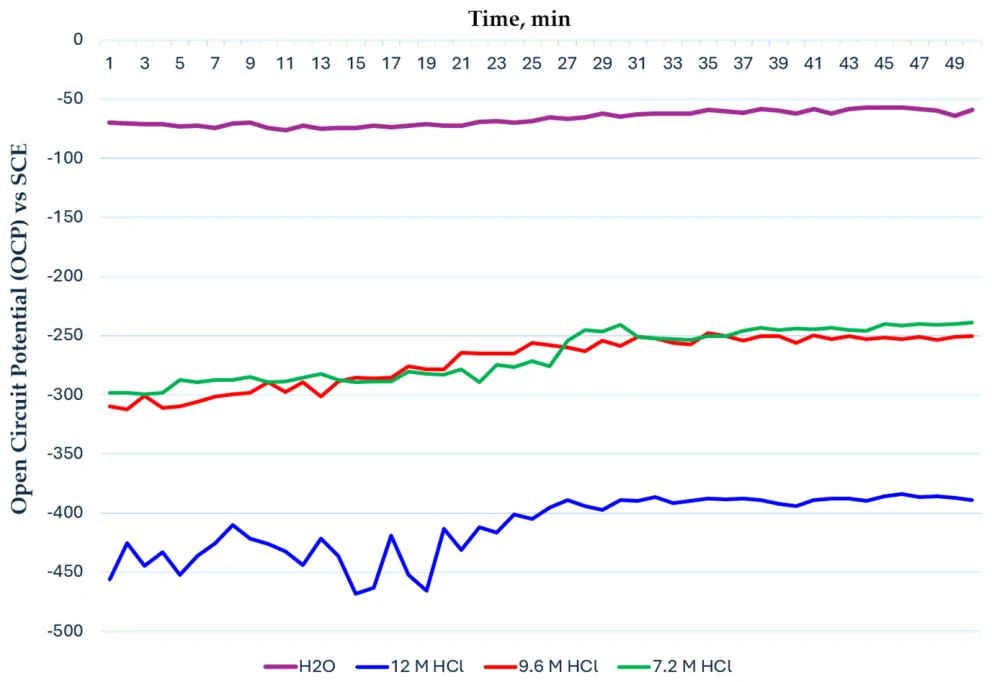
Open circuit potential (OCP) evolution of AISI 316L stainless steel during the 50 min stabilization period in tap water and HCl solutions of different concentrations.

Figure 3 shows the evolution of the open circuit potential (OCP) of AISI 316L stainless steel during the 50 min stabilization period prior to cyclic potentiodynamic polarization. The specimen immersed in tap water exhibited the most noble potential, stabilizing at approximately −60 mV vs. SCE, whereas the HCl solutions exhibited significantly more negative potentials. The most active potential was recorded in 12 M HCl (approximately −390 mV vs. SCE), while intermediate values were observed in 7.2 and 9.6 M HCl (approximately −240 to −250 mV vs. SCE). In all HCl solutions, the OCP gradually shifted toward more noble values during the stabilization period before reaching a nearly constant value, indicating that electrochemical equilibrium was attained prior to polarization testing. The progressive displacement of the OCP toward more negative values with increasing HCl concentration reflects a more active surface condition, consistent with the higher anodic dissolution rates observed during the subsequent cyclic potentiodynamic polarization tests.

After the OCP stabilization period, cyclic potentiodynamic polarization tests were performed to evaluate the corrosion behavior of AISI 316L stainless steel under the different exposure conditions.

#### 3.3.2. Cyclic Potentiodynamic Polarization

The kinetic behavior of corrosion in AISI 316L austenitic stainless steel was determined using cyclic potentiodynamic polarization and can be observed through cathodic and anodic reactions in the polarization curves. The Tafel extrapolation technique was used in the potentiodynamic polarization curves to determine the corrosion current density (I_corr_, µA·cm^2^), the corrosion potential (E_corr_, V), passivation current density (i_pass_), pitting potential (E_pit_), and the corrosion rate [5,45]. The corrosion current density was determined within a range of ±300 mV in the linear section of the cathodic and anodic branches of the polarization curves over at least one decade of current [45,49,50,51,52,53,54,55,56,57].

**Figure 4:**
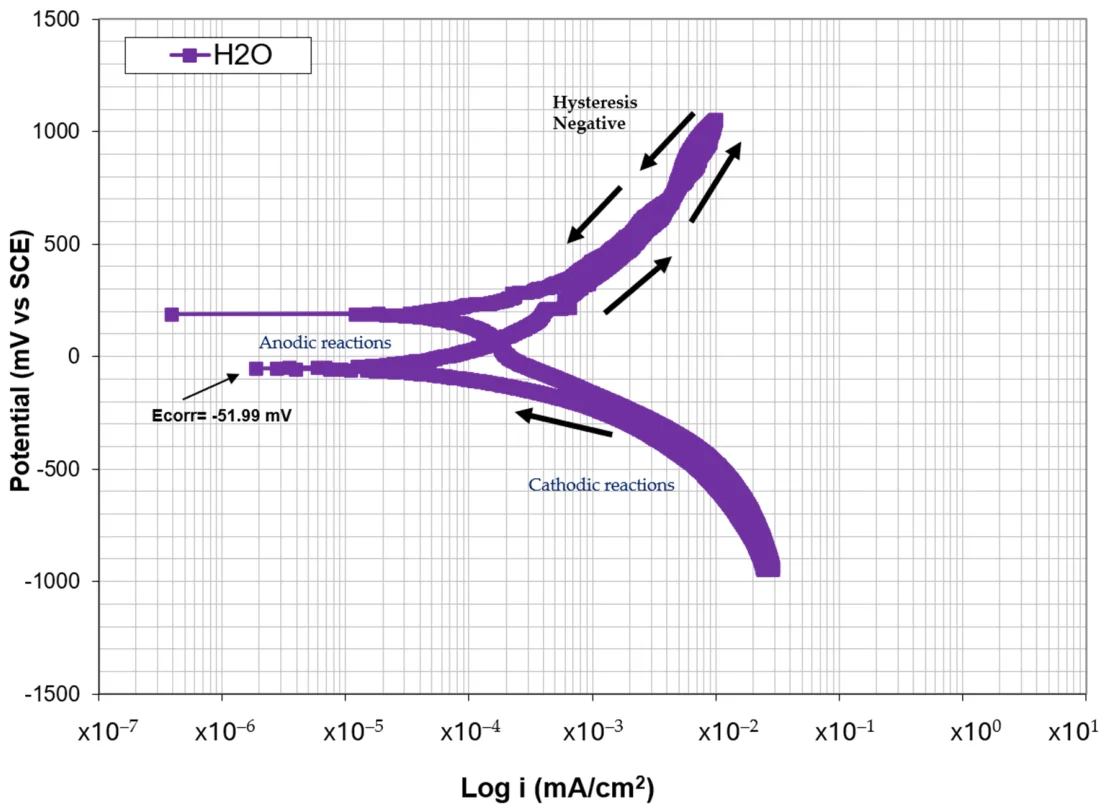
Cyclic potentiodynamic polarization curves of AISI 316L SS samples exposed in H_2_O solutions.

**Figure 5:**
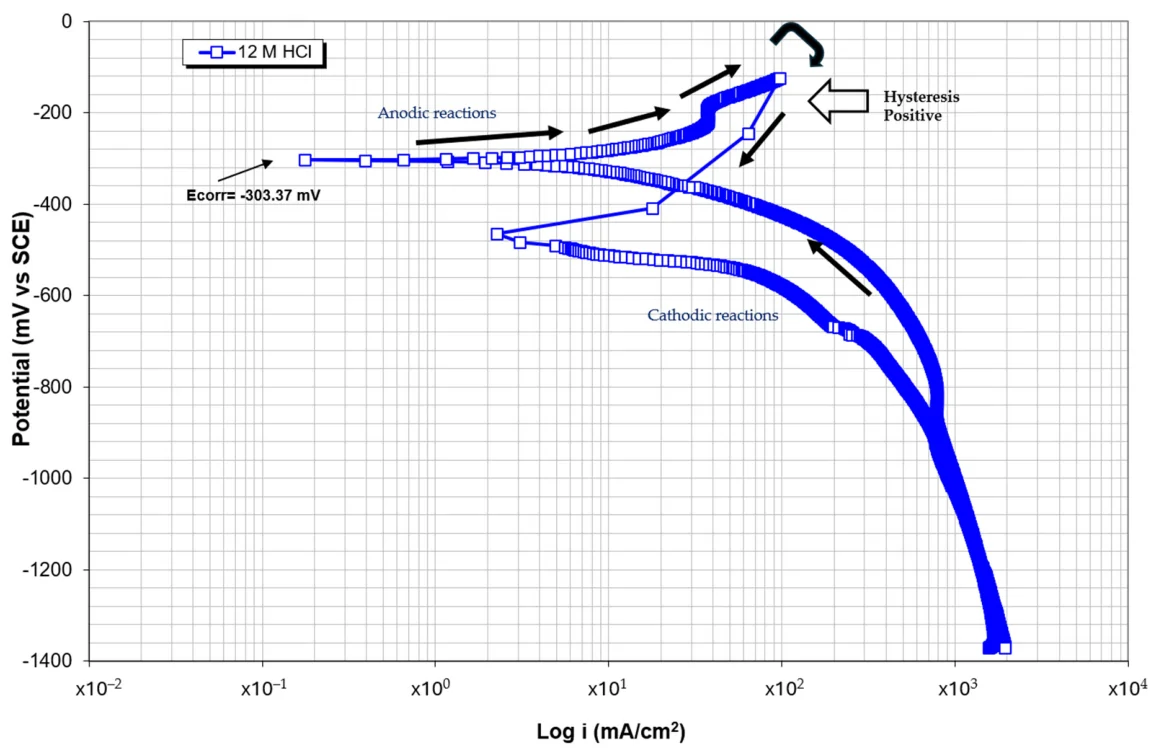
Cyclic potentiodynamic polarization curves of AISI 316L SS samples exposed in HCl (12 M) solutions.

**Figure 6:**
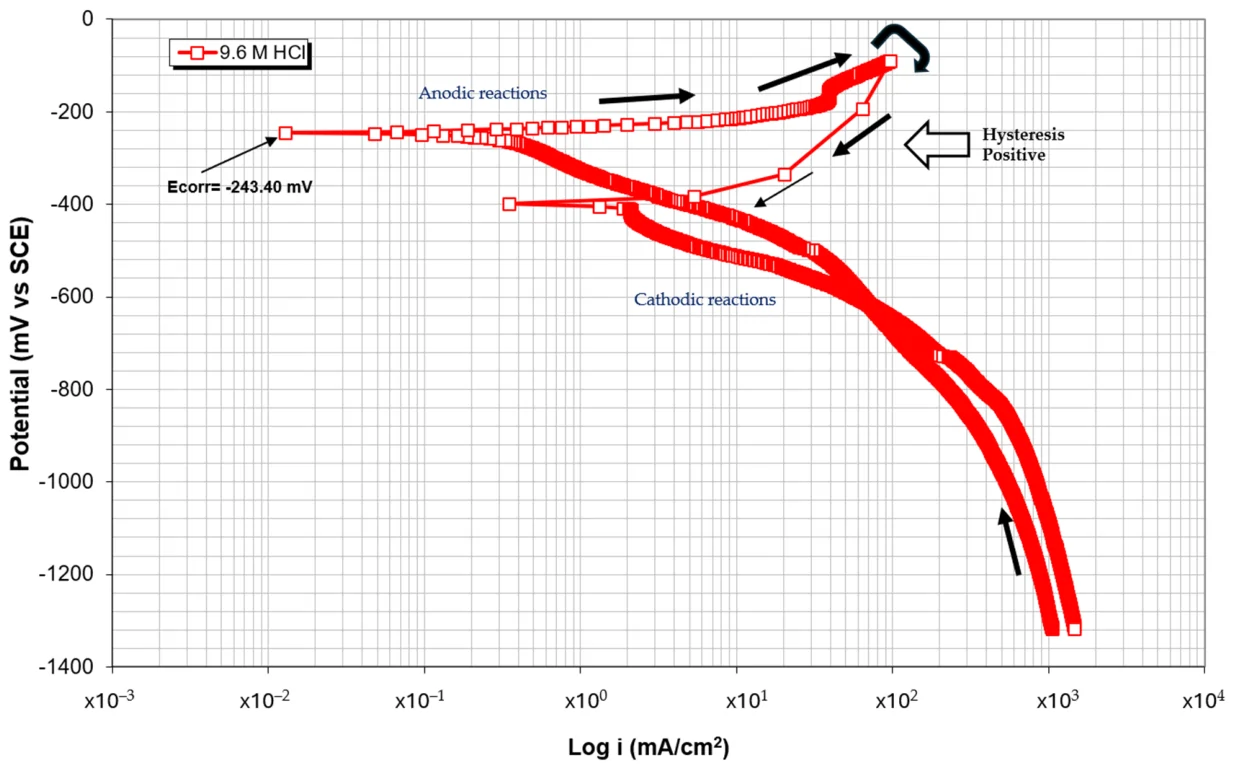
Cyclic potentiodynamic polarization curves of AISI 316L SS samples exposed in HCl (9.6 M) solutions.

**Figure 7:**
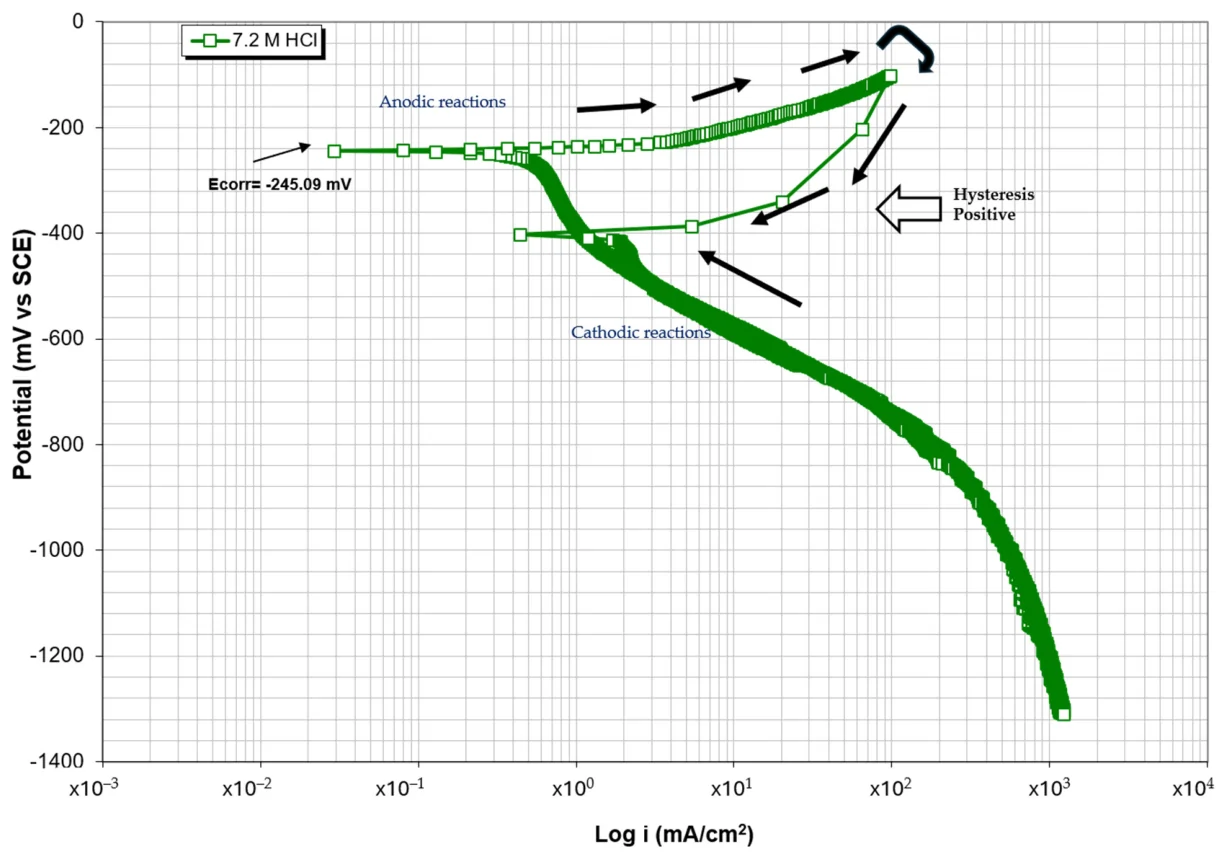
Cyclic potentiodynamic polarization curves of AISI 316L SS samples exposed in HCl (7.2 M) solutions.

The CPP curves obtained for AISI 316L stainless steel in the presence of different concentrations of hydrochloric acid and tap water as a non-aggressive solution are shown in Figure 4, Figure 5, Figure 6 and Figure 7. The CPP curve results indicate that the anodic and cathodic reactions of all systems showed activation in the anodic branch. No stable or extensive passive regions were observed (since the electrolyte at these concentrations is highly aggressive), and the cathodic branch was cathodically controlled, meaning that the corrosion rate now depends on the speed of the hydrogen reaction. If the reaction is slow, even though the metal is dissolving, the corrosion of the system is limited by the rate of the cathodic reaction.

Figure 4 presents the cyclic potentiodynamic polarization curve of AISI 316L stainless steel in water. The polarization behavior exhibits activation-controlled anodic and cathodic branches that define the mixed corrosion potential (E_corr_). A negative hysteresis loop was observed during the reverse scan, indicating effective repassivation and a low susceptibility to stable localized corrosion. This behavior is consistent with predominantly uniform corrosion under these conditions. The electrochemical parameters yielded a corrosion potential (E_corr_) of −51.99 mV and a corrosion current density on the order of 10^−4^ mA/cm^2^, confirming the excellent corrosion resistance of AISI 316L stainless steel in water.

Figure 5, Figure 6 and Figure 7 show the behavior of the cathodic and anodic reactions of AISI 316L SS steel in the presence of HCl at different concentrations, where the system is governed by the cathodic reaction, while in the anodic reaction, only activation and a small passivation of approximately 100 mV were observed for the concentrations of 12 M and 9.6 M, followed by a transfer-passivation.

The cyclic polarization curve obtained in AISI 316L SS exposed to different concentrations [12, 9.6, and 7.2 M] of hydrochloric acid solution showed predominantly active behavior, characterized by the absence of a stable passive region and a continuous increase in anodic current density. The wide hysteresis observed during the reverse scan is mainly attributed to surface morphological changes caused by the intense dissolution of the stainless steel, rather than to a classic pitting initiation and propagation process. Under these conditions, the test reflects the active dissolution kinetics of the material in a highly acidic medium, where the passive film rapidly destabilizes.

The results for AISI 316L stainless steel in the presence of hydrochloric acid at different concentrations show that the E_corr_ value is more active than the potential value in water; the values are more negative as the hydrochloric acid concentration increases (E_corr_ of −245.09 mV (7.2 M), −243.40 mV (9.6 M), and −303.37 mV (12 M)). Regarding the corrosion current density, i_corr_, for AISI 316L stainless steel in the presence of HCl at concentrations of 7.2 M and 9.6 M, it was on the order of ×10^−1^ mA/cm^2^. However, when 12 M HCl was present, the current density increased to the order of ×10^1^ mA/cm^2^.

All samples exhibited positive hysteresis (the loop is indicated in each of the polarization curves by dashed lines on the anodic branch), indicating a high susceptibility to active dissolution and evidence of non-formation of the passivation film.

**Figure 8:**
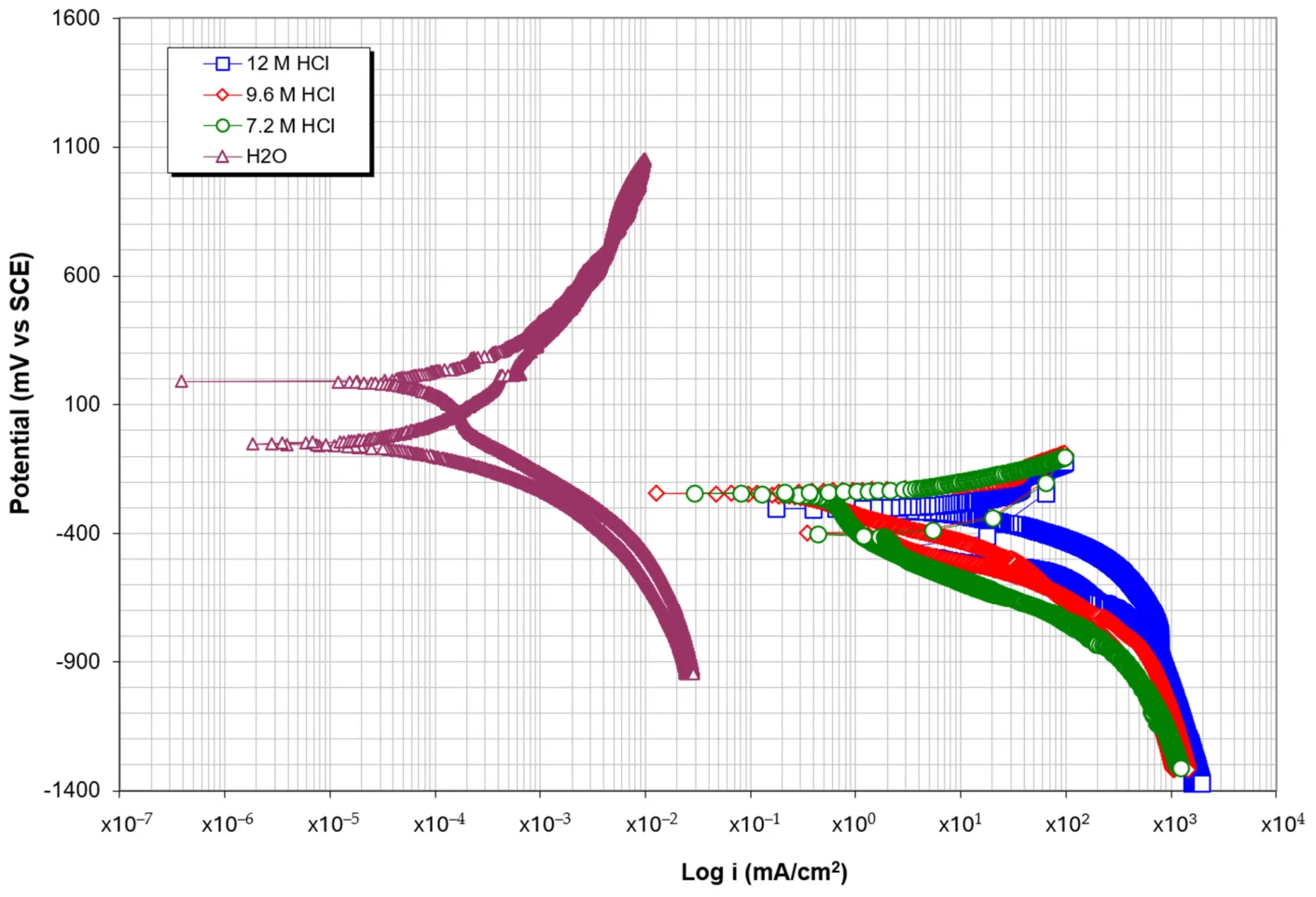
Cyclic potentiodynamic polarization curves of AISI 316L SS. All samples were exposed in H_2_O and HCl (12, 9.6 and 7.2 M) solutions.

In Figure 8, all the cyclic polarization curves of AISI 316L SS can be observed in the presence of water and the different concentrations of HCl. The shift of the polarization curves to the right represents a greater current demand as a response to the aggressiveness of the electrolyte.

Table 3 shows the electrochemical parameters obtained using cyclic potentiodynamic polarization (CPP) curves. The corrosion potential shifted markedly toward more negative values when AISI 316L stainless steel was exposed to concentrated HCl, confirming activation of the metallic surface and destabilization of the passive film. Cathodic Tafel slopes of approximately 117–126 mV dec^−1^ were obtained in water, 7.2 M HCl, and 12 M HCl, suggesting that proton reduction and hydrogen evolution remained the predominant cathodic process. However, the cathodic slope obtained in 9.6 M HCl and the anodic slope obtained in 12 M HCl were not considered mechanistically reliable because the corresponding branches did not exhibit sufficiently extensive linear Tafel regions. Consequently, these values were used only as qualitative indicators and were not employed to calculate absolute corrosion rates.

### 3.4. SEM After Electrochemical Measurements

**Figure 9:**
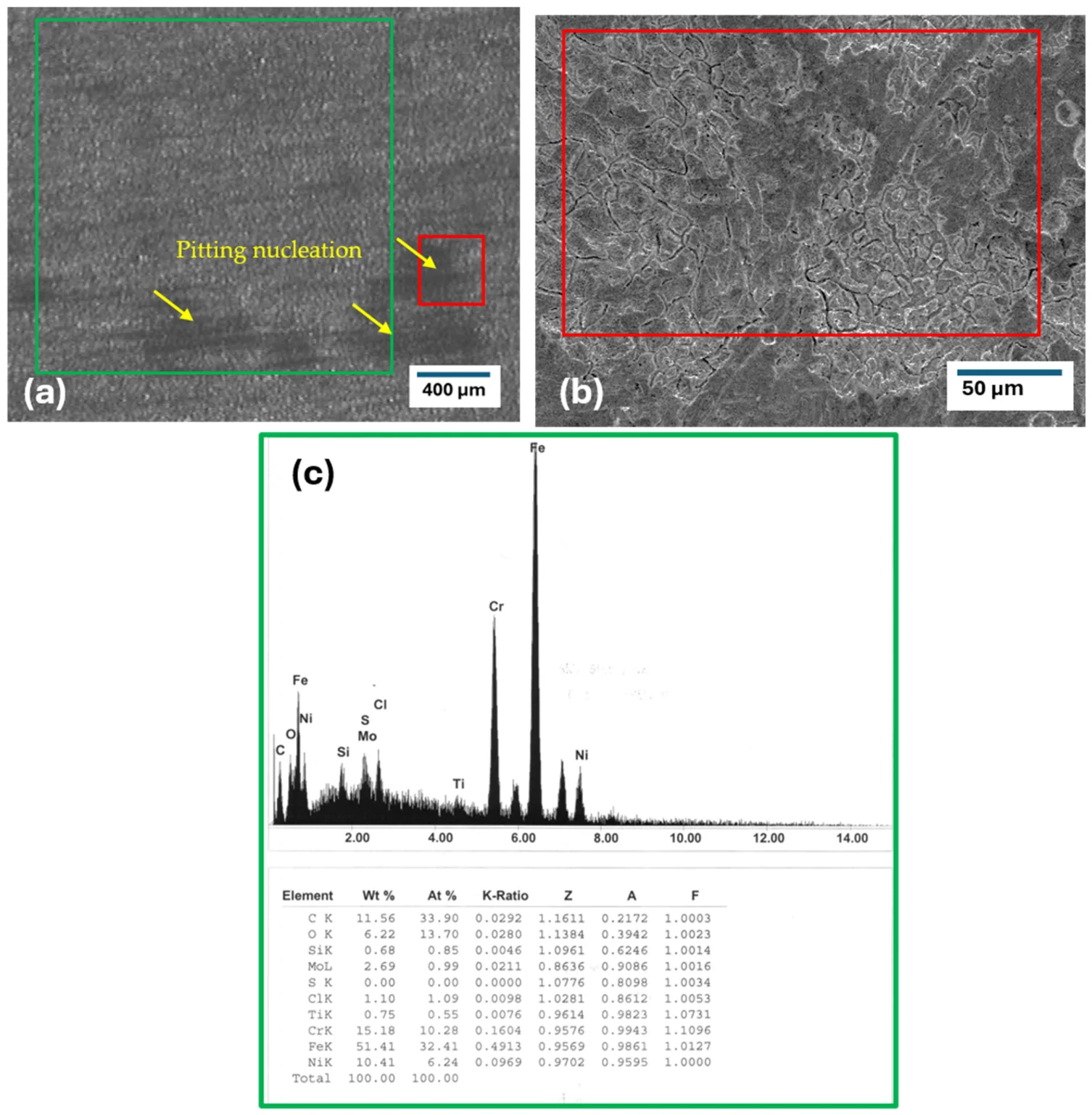
SEM-SE morphology for AISI 316L SS exposed to the H_2_O solution: (**a**) 55 x, (**b**) 1500 x, and (**c**) EDS spectrum.

Following the electrochemical tests, the corroded surfaces were characterized by scanning electron microscopy (SEM). The SEM micrograph of AISI 316L stainless steel exposed to tap water (Figure 9a) showed only a few dark surface features (green and red boxes), which may correspond to incipient localized surface attack or the early stages of pit initiation. At higher magnification (Figure 9b), only a limited amount of surface deposits was observed, indicating that the surface remained largely intact after exposure. Figure 9c presents the corresponding EDS spectrum, which identified the characteristic alloying elements of AISI 316L stainless steel (Fe, Cr, Ni, Mo, and Si), together with oxygen and a weak chlorine signal. The low chlorine content suggests limited chloride deposition from the aqueous environment and is consistent with the excellent corrosion resistance of AISI 316L stainless steel under these conditions.

**Figure 10:**
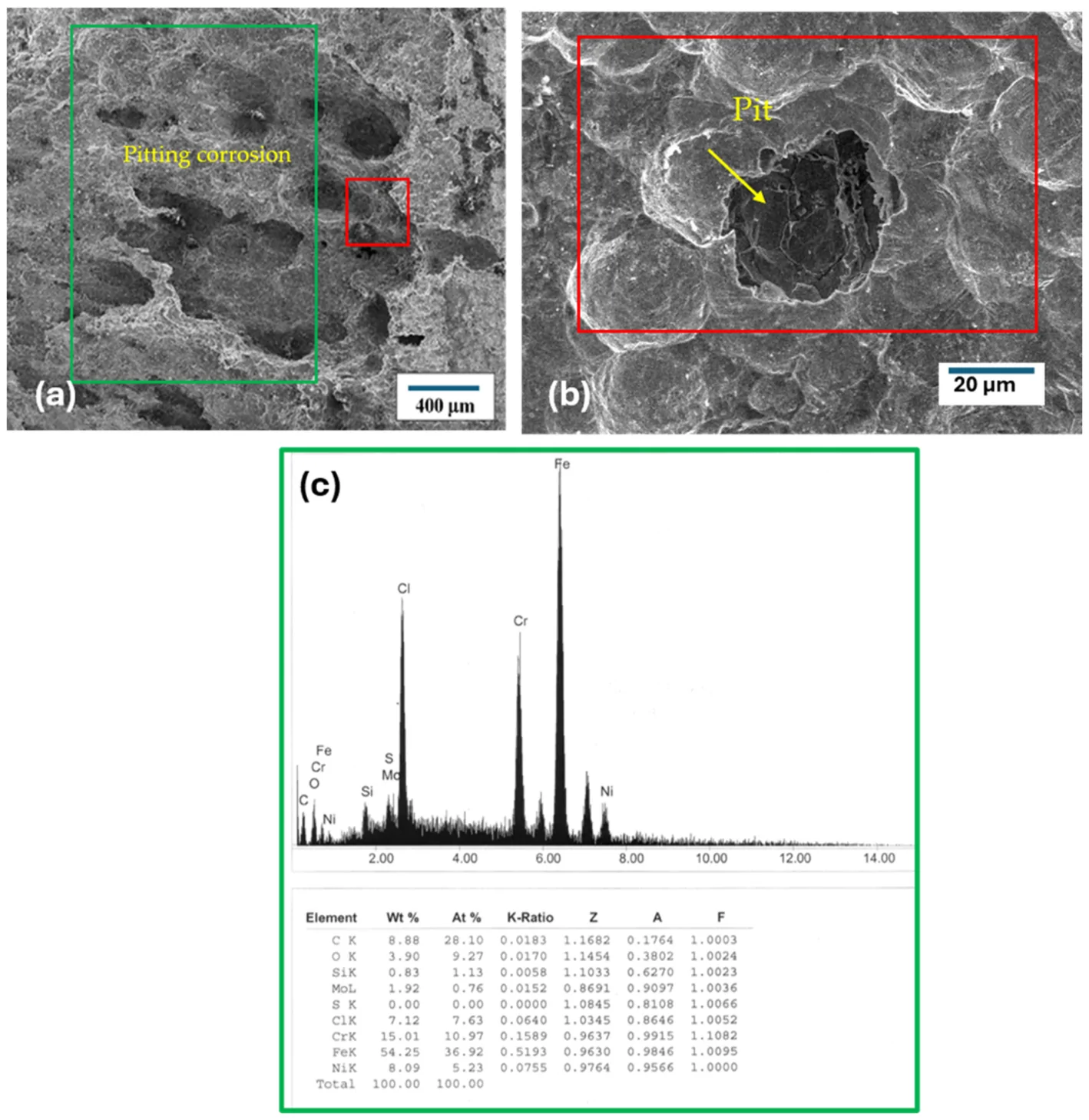
SEM-SE morphology for AISI 316L SS exposed to 12 M HCl solution. (**a**) 55 x, (**b**) 1500 x, and (**c**) EDS spectrum.

**Figure 11:**
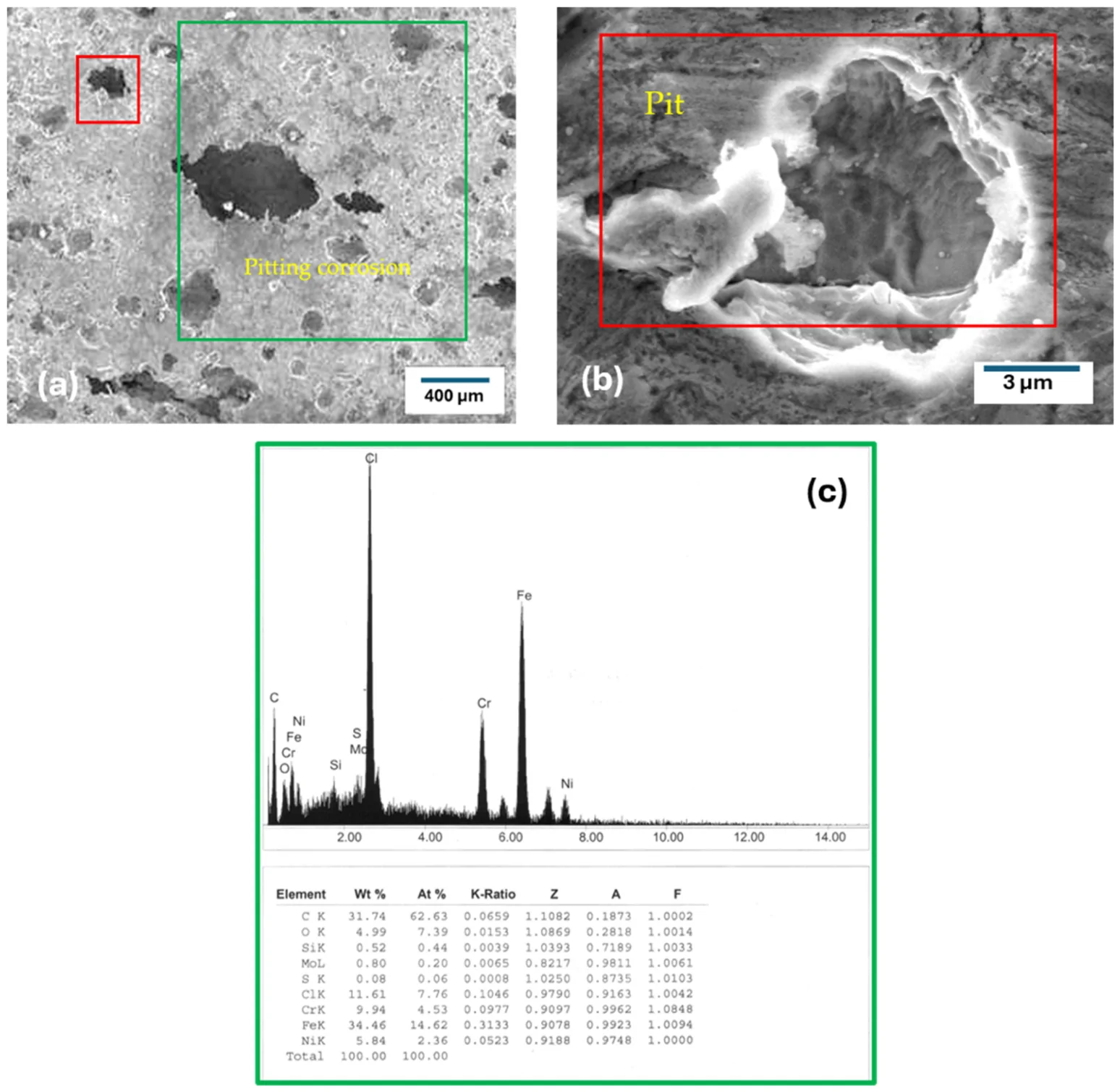
SEM-SE morphology for AISI 316L SS exposed to (**b**) 9.6 M solutions. (**a**) 55 x, (**b**) 1500 x, and (**c**) EDS spectrum.

**Figure 12:**
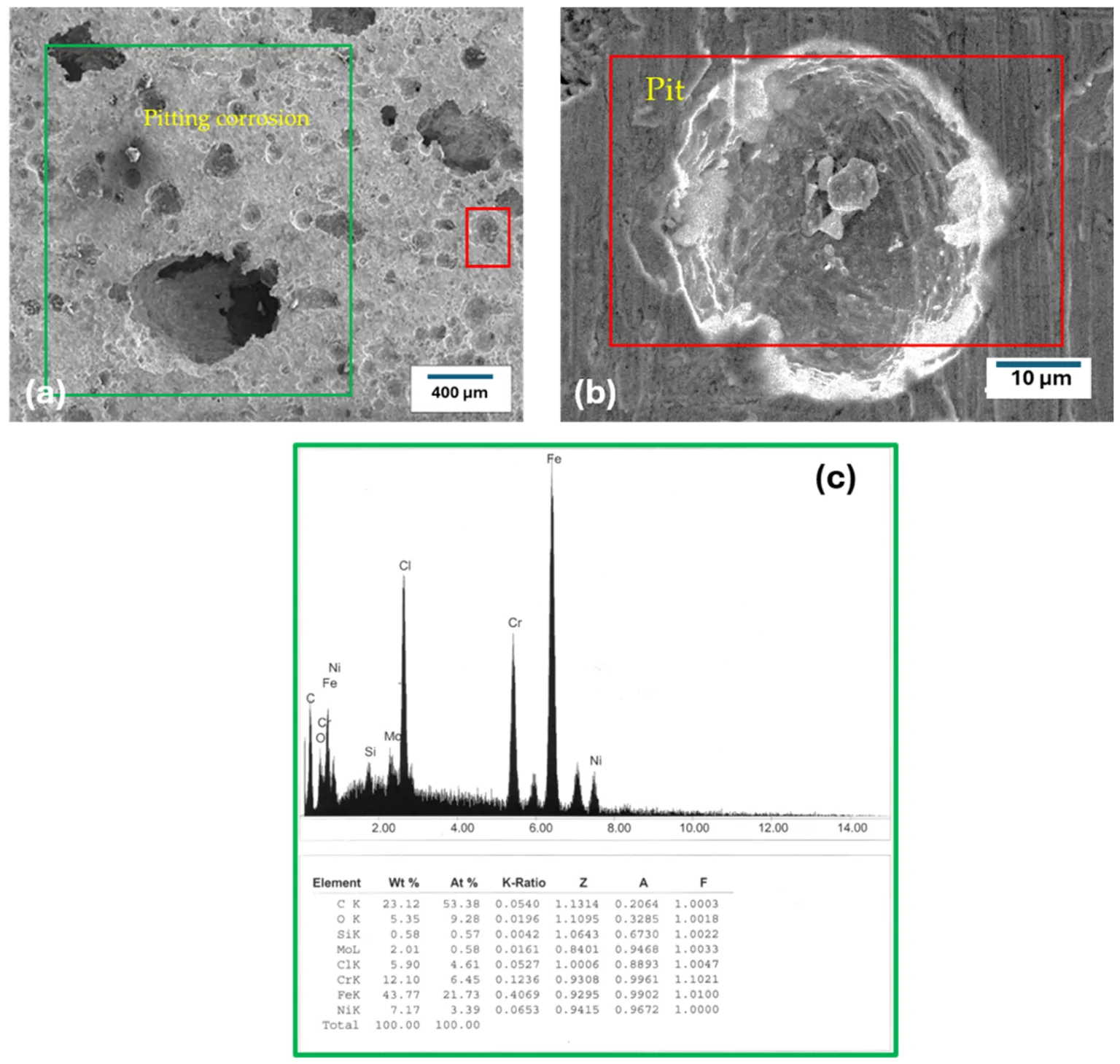
SEM-SE morphology for AISI 316L SS exposed to (**c**) 7.2 M HCl solution. (**a**) 55 x, (**b**) 1500 x, and (**c**) EDS spectrum.

The surface morphologies of AISI 316L stainless steel exposed to hydrochloric acid solutions of 12, 9.6, and 7.2 M are presented in Figure 10a, Figure 11a and Figure 12a, respectively. In all cases, localized pitting corrosion with pit diameters exceeding 400 µm was observed, confirming the severe degradation produced by the highly aggressive acidic environment. Higher-magnification SEM images of representative pits are shown in Figure 10b, Figure 11b and Figure 12b, corresponding to the regions highlighted by the red boxes in Figure 10a, Figure 11a and Figure 12a. The morphology of the pits revealed extensive localized dissolution associated with passive film breakdown. Figure 10c, Figure 11c and Figure 12c present the EDS spectra acquired from the regions indicated by the green boxes in Figure 10a, Figure 11a and Figure 12a. The elemental analysis identified Fe, Cr, Ni, Mo, Si, and O, together with a significant chlorine signal. The presence of chlorine on the corroded surface is consistent with the adsorption of chloride-containing species, which are known to contribute to passive film destabilization and localized corrosion in stainless steels.

**Figure 13:**
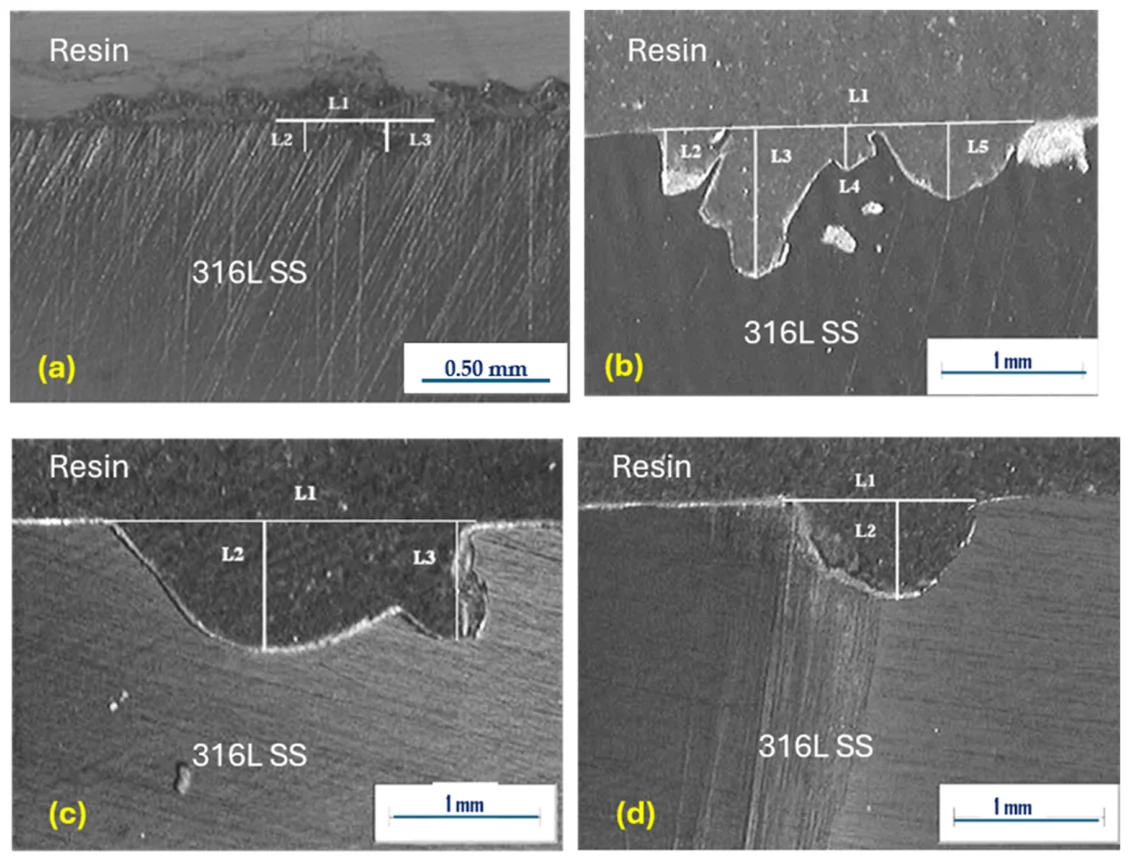
OM stereoscope morphology for AISI 316L SS exposed to H_2_O (**a**), 12 M HCl (**b**), 9.6 M (**c**) HCl, and 7.2 M HCl (**d**) solutions.

Following SEM surface characterization, transverse cross-sections were prepared to provide a complementary evaluation of the morphology and penetration of representative pits formed on AISI 316L stainless steel after exposure to the different HCl solutions. Representative cross-sections are shown in Figure 13. The pit dimensions were measured using the parameters L1 (surface opening), L2 (pit penetration depth), and the additional geometric measurements summarized in Table 4. Because one representative cross-section was examined for each exposure condition, the reported dimensions are descriptive measurements intended to illustrate the pit morphology and penetration and should not be interpreted as a statistical characterization of the entire corroded surface.

The specimen exposed to water exhibited only slight surface degradation with shallow cavities not exceeding 0.2 mm in depth, indicating minimal localized attack. In contrast, all HCl solutions produced well-defined pits extending beneath the original surface. The deepest penetration (0.874 mm) was measured for the 9.6 M HCl solution, whereas the 12 M solution produced wider cavities with a maximum depth of 0.44 mm. The specimen exposed to 7.2 M HCl exhibited an intermediate behavior, with a pit depth of 0.64 mm. These observations indicate that exposure to concentrated HCl promotes severe localized dissolution; however, the deepest pits were not necessarily associated with the highest acid concentration. At 12 M HCl, the higher overall dissolution rate appears to favor lateral propagation and the coalescence of neighboring pits, whereas at 9.6 M HCl, localized dissolution remained sufficiently stable to promote deeper penetration into the substrate [58,59,60].

It should be noted that the SEM-EDS analysis provided information on the morphology and elemental composition of the corroded surfaces. The specific chemical nature of the surface species was not determined in the present study, and therefore no definitive identification of individual corrosion products is made.

The cross-sectional morphology agrees with the pit geometries described in ASTM G46-21, exhibiting wide subsurface cavities with approximately hemispherical to elliptical profiles and significant penetration beneath the original surface [61].

The reported dimensions were obtained from the individual representative cross-sections shown in Figure 13. The values are descriptive measurements and do not represent statistical averages or the maximum pit dimensions over the entire specimen surface.

**Figure 14:**
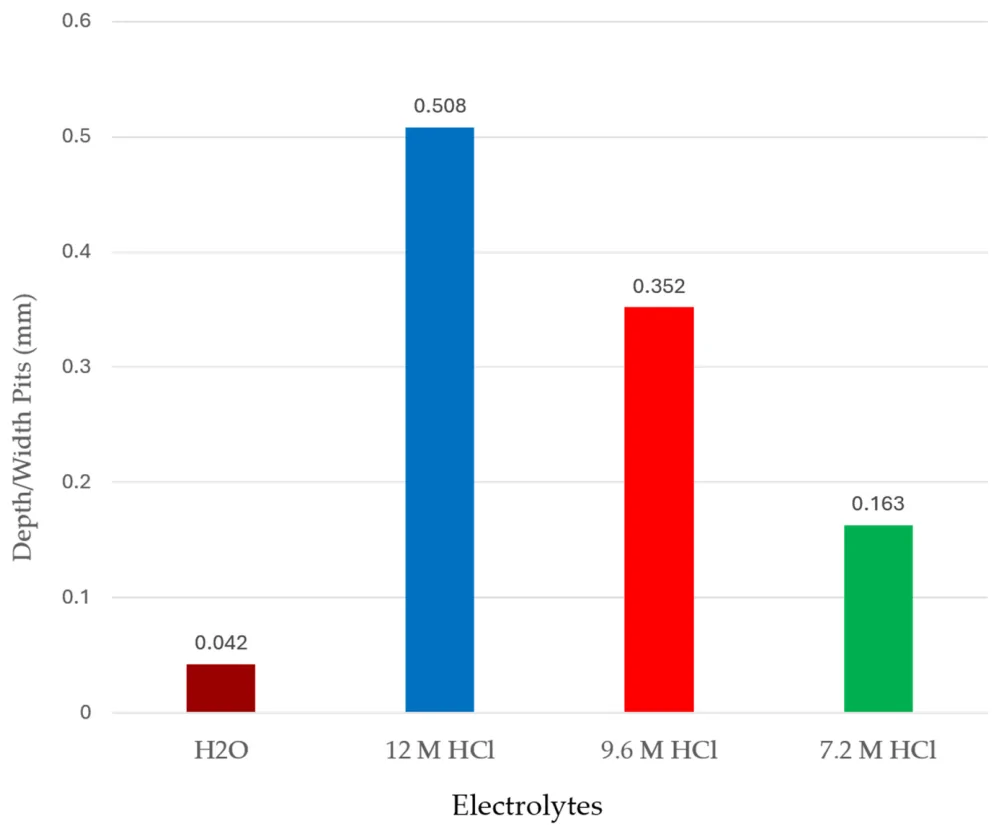
Pit depth measured from representative metallographic cross-sections as a function of HCl concentration.

Figure 14 compares the pit depths measured from the representative metallographic cross-sections as a function of HCl concentration. The results indicate that the measured pit depth did not increase monotonically with acid concentration. Although the 12 M HCl solution produced the highest corrosion current densities during electrochemical testing, the greatest pit depth measured from the representative cross-sections was observed for the 9.6 M HCl solution (0.874 mm). This behavior suggests that the morphology and propagation of localized attack depend not only on acid concentration, but also on the balance between localized pit growth and general active dissolution. At 9.6 M HCl, the electrochemical conditions appear to favor sustained localized dissolution, promoting deeper pit propagation into the substrate. In contrast, exposure to 12 M HCl resulted in significantly higher overall dissolution rates, which may have favored lateral pit growth and the coalescence of neighboring pits rather than continued penetration into the substrate [62]. Because these measurements were obtained from one representative metallographic cross-section for each exposure condition, the results should be interpreted as descriptive observations rather than as a statistical characterization of pit-depth distribution over the entire specimen surface.

Because the pit-depth measurements were obtained from one representative metallographic cross-section for each exposure condition, the reported values should be interpreted as descriptive measurements of the selected pits rather than as a statistical characterization of the pit-depth distribution over the entire corroded surface. Nevertheless, these observations provide useful complementary information to the electrochemical and SEM analyses regarding the morphology and penetration of localized corrosion.

AISI 316L stainless steel is widely used in ISO tanks and process equipment in the petrochemical industry because of its favorable combination of corrosion resistance and mechanical properties. Nevertheless, exposure to concentrated hydrochloric acid (HCl), which may occur during polymer production, cleaning operations, or contact with acidic process residues, can severely compromise its corrosion resistance. The combined action of hydrogen ions (H^+^) and chloride ions (Cl^−^) destabilizes the chromium-rich passive film that normally protects the alloy, thereby promoting active dissolution and localized corrosion. Under these highly aggressive conditions, the protective film cannot be effectively regenerated because its dissolution rate exceeds its formation rate, resulting in continued degradation of the metallic surface [63,64,65].

Recent investigations on stainless steels exposed to hydrochloric acid have primarily addressed passive-film degradation, localized corrosion mechanisms, corrosion inhibition, alloy development, and the use of duplex and superduplex stainless steels in aggressive environments. However, comparatively few studies have examined the active dissolution behavior of conventional AISI 316L stainless steel in highly concentrated HCl solutions relevant to severe industrial exposure conditions. Superduplex stainless steels, with PREN values typically ranging from 40 to 43, generally exhibit greater resistance to localized corrosion than AISI 316L stainless steel, for which a PREN value of 24.5 was calculated in the present study. Although PREN is useful for comparing the intrinsic resistance of stainless steels to localized corrosion, it cannot independently predict their performance in strongly acidic media. The PREN expression does not account for factors such as passive-film stability, surface condition, nonmetallic inclusions, microstructural heterogeneity, elemental segregation resulting from solidification or welding, temperature, and electrolyte composition. Therefore, electrochemical testing remains essential for evaluating corrosion behavior under specific exposure conditions [48,49,66].

AISI 316L stainless steel undergoes active dissolution when its chromium-rich passive film becomes unstable because its dissolution rate exceeds its regeneration rate under highly aggressive environments, such as concentrated hydrochloric acid solutions. The combined action of hydrogen ions (H^+^) and chloride ions (Cl^−^) accelerates passive film degradation, preventing the establishment of stable passivity. Under these conditions, the alloy no longer behaves as a passive material, resulting in a significant increase in the corrosion rate.

The progressive displacement of the OCP toward more negative values with increasing HCl concentration is consistent with the corrosion potentials (Ecorr) obtained from the cyclic polarization curves. Although OCP and Ecorr are not identical electrochemical parameters, both exhibited the same tendency, indicating increasingly active surface conditions as the aggressiveness of the electrolyte increased. This behavior agrees with the higher corrosion current densities measured in concentrated HCl solutions and supports the transition toward an active dissolution regime.

The cyclic potentiodynamic polarization curves of AISI 316L stainless steel (Figure 5, Figure 6 and Figure 7) exhibited similar electrochemical behavior, characterized by active dissolution throughout the anodic polarization region. No well-defined passive region was observed, indicating that a stable protective passive film was not established under the investigated conditions. This electrochemical response is consistent with the highly aggressive nature of concentrated HCl solutions, where continuous passive-film degradation predominates over film regeneration, thereby facilitating sustained metal dissolution and localized corrosion [67,68,69,70].

Under normal service conditions, stainless steels are protected by a thin chromium-rich passive film that provides excellent corrosion resistance [65]. However, in concentrated hydrochloric acid solutions, the combined action of hydrogen and chloride ions destabilizes this protective film, promoting continuous active dissolution and facilitating localized corrosion. The severity of this degradation generally increases with increasing acid concentration and temperature, reducing the ability of the alloy to maintain its passive state.

The electrochemical response indicates that the corrosion behavior of AISI 316L stainless steel is dominated by active dissolution, as evidenced by the absence of a well-defined passive region in the cyclic potentiodynamic polarization curves. However, scanning electron microscopy revealed that this dissolution was not completely uniform across the exposed surface. Instead, deep cavities exhibiting characteristic localized corrosion morphology were observed, indicating that localized attack develops simultaneously with generalized active dissolution. This behavior suggests that the highly aggressive HCl solutions rapidly destabilize the passive film over the entire surface while promoting preferential dissolution at microstructurally more susceptible sites, resulting in the formation of deep localized cavities. Consequently, the degradation mechanism of AISI 316L stainless steel in concentrated HCl should be interpreted as the combined action of generalized active dissolution and accelerated localized dissolution. The electrochemical response describes the overall corrosion behavior, whereas SEM observations reveal the preferential development of localized damage responsible for the final surface morphology [59,63,64,65,66,67,68,69].

**Figure 15:**
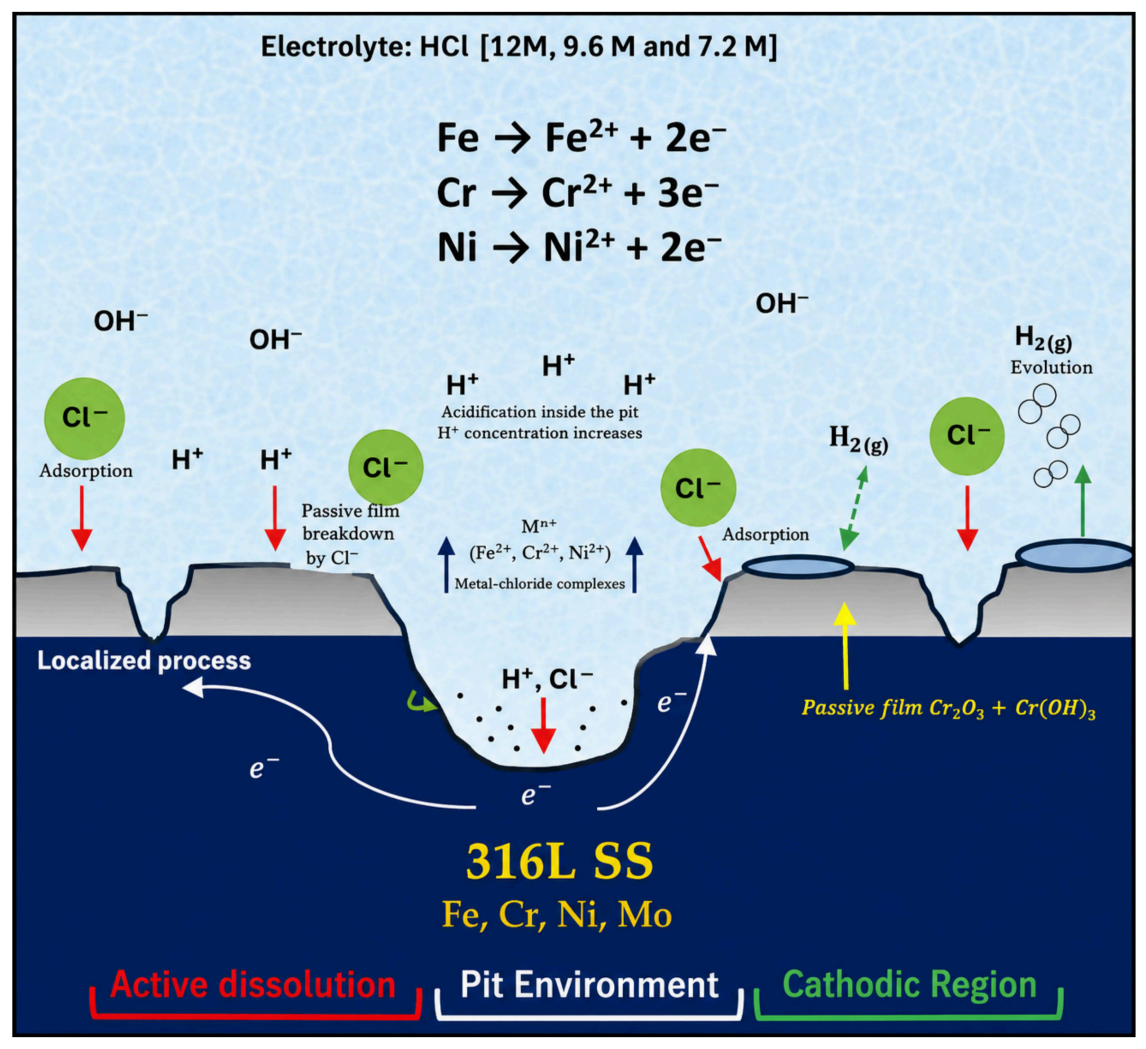
Diagram of the mechanism of the combination of generalized active dissolution and accelerated localized dissolution of AISI 316L SS in the presence of HCl solution.

Based on the electrochemical and microstructural observations, a corrosion mechanism is proposed for AISI 316L stainless steel in concentrated HCl solutions. The electrochemical reactions involved in this degradation process are summarized in Equations (2)–(11), while Figure 15 schematically illustrates the combined mechanism of generalized active dissolution and accelerated localized dissolution [70,71,72,73,74,75].

#### Anodic Dissolution

Fe → Fe^2+^ + 2e^−^ (2)

Cr→Cr^3+^ + 3e^−^ (3)

(4) $$Ni\to Ni^{2+}+2e^{-}$$

2H^+^ + 2e^−^ → H_2(g)_     cathodic reaction (5)

M + nH^+^ → M^n+^ + n/2H_2(g)_ Overall electrochemical reaction (6)

where M represents Fe, Cr, or Ni, and n corresponds to the oxidation state of the dissolving metal.

HCl_(aq)_ → H^+^_(aq)_ + Cl^−^_(aq)_ dissociation of hydrochloric acid (7)

Possible formation of soluble metal chloride complexes:

Fe^2+^ + 2CL^−^ ⇌ FeCl_2(aq)_ (8)

Cr^2+^ + 3Cl^−^ ⇌ CrCl_3(aq)_ (9)

Ni^2+^ + 2Cl^−^ ⇌ NiCl_2(aq)_ (10)

M^n+^ + H_2_ O→MOH^(n−1)+^ + H^+^ hydrolysis of dissolved metal ions (11)

The corrosion behavior in the present study was evaluated by cyclic potentiodynamic polarization in accordance with ASTM G61 and correlated with post-corrosion microstructural observations. Although complementary gravimetric measurements could provide average corrosion rates over longer exposure periods, such measurements were beyond the scope of the present investigation and represent an interesting topic for future work.

## 4. Conclusions

This work investigated the corrosion behavior of AISI 316L austenitic stainless steel exposed to concentrated hydrochloric acid (HCl) solutions (7.2, 9.6, and 12 M) at room temperature by cyclic potentiodynamic polarization and microstructural characterization. The main conclusions are as follows:

- The PREN value (24.5) indicates the intrinsic resistance of AISI 316L stainless steel to localized corrosion under passive conditions. However, in concentrated HCl solutions, the aggressive electrolyte destabilizes the passive film, substantially reducing the protective effect associated with the alloy composition.
- The open-circuit potential shifted progressively toward more negative values with increasing HCl concentration, consistent with the corrosion potentials obtained from cyclic polarization. This behavior indicates increasingly active surface conditions prior to polarization and agrees with the higher corrosion current densities associated with active dissolution.
- The corrosion current density increased by approximately two orders of magnitude when the HCl concentration increased from 7.2–9.6 M to 12 M, demonstrating a significant acceleration of the active dissolution kinetics.
- The cyclic polarization curves exhibited an active anodic dissolution regime without a stable passive region, indicating that concentrated HCl prevents the establishment and regeneration of a protective chromium-rich passive film. The cathodic reaction was dominated by hydrogen evolution.
- Positive hysteresis loops were observed for all HCl concentrations, indicating irreversible surface damage during anodic polarization. SEM observations confirmed that localized corrosion developed simultaneously with generalized active dissolution, producing pits larger than 400 µm in diameter.
- The corrosion mechanism of AISI 316L stainless steel in concentrated HCl is governed by the combined action of generalized active dissolution and accelerated localized dissolution. Active dissolution dominates the overall electrochemical response, whereas localized attack determines the morphology and severity of the surface damage.
- Increasing HCl concentration progressively destabilized the passive film, resulting in higher corrosion current densities, lower corrosion resistance, and greater susceptibility to localized corrosion.

## Figures and Tables

**Table 1:** Chemical composition of AISI 316L stainless steel (wt.%).

Material (SS)	C	Cr	Ni	Mo	Mn	Si	S	P	N	Fe
AISI 316L	0.030 max	15.57–17.20	9.49–10.59	2.0–2.20	1.38–1.53	0.62–0.69	0.19–0.21	0.089–0.094	0.05	Balance

**Table 2:** Pitting resistance equivalent numbers of the AISI 316L stainless steels.

Sample	Cr	Mo	N	PREN
**AISI 316L**	16.77	2.10	0.05	24.5

**Table 3:** Parameters obtained by CPP for AISI 316L stainless steel evaluated in HCl solution.

Solution	E_corr_ (mV vs. ESC)	I_corr_ (mA/cm^2^)	βa (mV dec^−1^)	βc (mV dec^−1^)	Hysteresis	Corrosion Mechanism
H_2_O	−51.99	4.8 × 10^−4^	177.4	118.4	Negative	Passive/uniform corrosion
12 M HCl	−303.37	1.2 × 10^1^	332.2 *	125.5	Positive	Active dissolution + pitting
9.6 M HCl	−243.40	2.6 × 10^−1^	98.8	529.7+	Positive	Active dissolution + pitting
7.2 M HCl	−245.09	2.4 × 10^−1^	205.5	117.4	Positive	Active dissolution + pitting

* Value considered approximate because a well-defined Tafel region was not obtained.

**Table 4:** Representative pit dimensions measured from metallographic cross-sections of AISI 316L stainless steel exposed to HCl solutions.

Solution	Pit Depth Measured in the Representative Cross-Section (mm)				
	L1	L2	L3	L4	L5
H_2_O	4.71	0.11	0.11	−	−
12 M HCl	2.70	0.44	1.05	0.30	0.53
9.6 M HCl	2.48	0.87	0.78	−	−
7.2 M HCl	1.26	0.64	−	−	−

## Data Availability

The original contributions presented in this study are included in the article. Further inquiries can be directed to the corresponding authors.
